# Equity-Oriented Monitoring in the Context of Universal Health Coverage

**DOI:** 10.1371/journal.pmed.1001727

**Published:** 2014-09-22

**Authors:** Ahmad Reza Hosseinpoor, Nicole Bergen, Theadora Koller, Amit Prasad, Anne Schlotheuber, Nicole Valentine, John Lynch, Jeanette Vega

**Affiliations:** 1 Department of Health Statistics and Information Systems, World Health Organization, Geneva, Switzerland; 2 Gender, Equity and Human Rights, World Health Organization, Geneva, Switzerland; 3 Centre for Health Development, World Health Organization, Kobe, Japan; 4 Department of Public Health, Environmental and Social Determinants of Health, World Health Organization, Geneva, Switzerland; 5 School of Population Health, University of Adelaide, Adelaide, Australia; 6 Rockefeller Foundation, New York, New York, United States of America

## Abstract

Monitoring inequalities in health is fundamental to the equitable and progressive realization of universal health coverage (UHC). A successful approach to global inequality monitoring must be intuitive enough for widespread adoption, yet maintain technical credibility. This article discusses methodological considerations for equity-oriented monitoring of UHC, and proposes recommendations for monitoring and target setting. Inequality is multidimensional, such that the extent of inequality may vary considerably across different dimensions such as economic status, education, sex, and urban/rural residence. Hence, global monitoring should include complementary dimensions of inequality (such as economic status and urban/rural residence) as well as sex. For a given dimension of inequality, subgroups for monitoring must be formulated taking into consideration applicability of the criteria across countries and subgroup heterogeneity. For economic-related inequality, we recommend forming subgroups as quintiles, and for urban/rural inequality we recommend a binary categorization. Inequality spans populations, thus appropriate approaches to monitoring should be based on comparisons between two subgroups (gap approach) or across multiple subgroups (whole spectrum approach). When measuring inequality absolute and relative measures should be reported together, along with disaggregated data; inequality should be reported alongside the national average. We recommend targets based on proportional reductions in absolute inequality across populations. Building capacity for health inequality monitoring is timely, relevant, and important. The development of high-quality health information systems, including data collection, analysis, interpretation, and reporting practices that are linked to review and evaluation cycles across health systems, will enable effective global and national health inequality monitoring. These actions will support equity-oriented progressive realization of UHC.

## Abstract 2

As part of the Universal Health Coverage Collection, Ahmad Reza Hosseinpoor and colleagues discuss methodological considerations for equity-oriented monitoring of universal health coverage, and propose recommendations for monitoring and target setting.

This paper is part of the PLOS Universal Health Coverage Collection.

**Summary Points**

- The equitable realization of universal health coverage requires an equity-oriented approach to monitoring; equity advocates should be unified in proposing a technically sound platform for monitoring that is easy to understand and communicate.
- Global monitoring should include complementary dimensions of inequality (such as economic status and urban/rural residence, in addition to sex), adopt a gap or whole spectrum approach, and conceptualize economic-related measures using quintiles.
- Both absolute and relative measures of inequality as well as disaggregated data should be reported, and national averages should be presented alongside inequality monitoring.
- Targets for global monitoring of health inequalities should be based on proportional reduction of absolute inequality.
- Countries can develop capacity for health inequality monitoring by strengthening health information systems for data collection, analysis, reporting, and dissemination.

## Background

In recent years the monitoring of health inequalities—defined as the observed health differences between subgroups of a population—has gathered momentum at the global level [1]–[4]. Monitoring health inequalities can be considered a platform for assessing health inequity—a normative concept referring to avoidable and unjust health differences between subgroups of a population, stemming from a form of social disadvantage [5]. Global monitoring of within-country health inequalities (i.e., cross-country comparisons of within-country inequalities based on standardized indicators and measurement approaches) is an important practice in the promotion of health equity, as it facilitates comparisons across borders and over time, and enables countries to perform benchmarking and learn from the experiences of one another [6]. Concurrent national-level inequality monitoring is valuable beyond its contribution to global monitoring to take into account context-specific factors and priorities.

As the end-date of the Millennium Development Goals draws nearer, plans for the post-2015 global development framework include a concentrated focus on universal health coverage (UHC) [7], a movement that is a longstanding and growing priority for the World Health Organization and its member states [8], and endorsed by the United Nations General Assembly [9]. The ultimate goal of UHC is directly linked to eliminating inequities: to ensure that all people who need health services are able to get them, without experiencing undue financial hardship [8],[10]. However, unless they are designed with an equity-oriented approach, movements toward UHC may facilitate early and/or accelerated gains for advantaged subgroups, while leaving others behind [11]. This “trickle down” implementation may worsen the situation for disadvantaged populations according to the inverse care law [12], and may exacerbate inequalities if universality is not fully achieved [13]. Thus, monitoring inequalities is fundamental to track the impact of health interventions that aim for universality, to ensure that the process leaves no disadvantaged group behind, and to promote concurrent or hastened progress among the most disadvantaged and across the social gradient [14].

Recommendations surrounding the post-2015 development agenda [7],[15] as well as UHC [8],[16],[17] have called for focused attention on monitoring the reduction of inequalities [8],[16],[17]. Indeed, the emerging global movement toward UHC presents opportunities for the widespread promotion and mainstreaming of health inequality monitoring at the global level. Advocates for health equity would be judicious to adopt a united front to rally for equity-related indicators and targets that are likely to be accepted and implemented by diverse stakeholders. Establishing methods and targets for global monitoring facilitates global comparisons that are meaningful and substantive ways of measuring and reporting progress in a set of common indicators.

Global monitoring of health inequality requires an overarching and unified approach. Global monitoring for UHC must be straightforward and easily understood while, at the same time, maintaining sufficient technical rigor to inform evidence-based decision making. Along with identifying a common set of health indicators across countries, consideration should be given to: selecting relevant dimensions of inequality (based on which dimension[s] will population subgroups be defined?), formulating subgroups (how should dimensions of inequality be defined to measure inequality between the disadvantaged and advantaged?), selecting appropriate approaches to monitoring (how will comparisons be made across populations?), measuring and communicating inequality (how can situations of inequality be expressed comprehensively?), and setting targets (how will success be measured?).

The objective of this paper is to review methodological considerations of monitoring health inequality, proposing recommendations for monitoring and target setting. If widely adopted and put into practice, a well-constructed protocol for global monitoring of within-country inequalities will bring stakeholders together with a clear and common purpose, catalyzing equity-oriented progress towards UHC.

## Relevant Dimensions of Inequality

Inequality is multidimensional, and it has been widely recommended that inequality monitoring include several diverse dimensions of inequality [6],[18],[19]. Monitoring multiple dimensions of inequality is not only conceptually important for capturing different axes of inequality, but has relevance on a practical level, as different dimensions of inequality often imply different interventions. Interventions that aim to improve economic-related inequality in health service coverage, for example, are unlikely to be the same as interventions to reduce sex-related health service coverage inequality.

Certain dimensions of inequality, such as sex and age, constitute important factors, but may not be applicable to all health indicators. For example, data should be disaggregated by sex whenever possible, noting that sex may not be a relevant dimension of inequality for indicators that only apply to one sex, such as female-specific reproductive health services and maternal health services. Age is only considered to be a relevant dimension of inequality when age discrimination yields inequitable health service coverage, as might be the case with contraceptive prevalence among adolescent versus adult women.

Previously, the Commission on Information and Accountability for Women's and Children's Health recommended the disaggregation of maternal and child health data according to six key dimensions (wealth, sex, age, urban/rural residence, geographical location, and ethnicity) as well as, where appropriate, education, marital status, number of children, and HIV status [19]. The World Health Assembly Resolution 62.14, endorsing the findings of the Commission on Social Determinants of Health, called on countries to disaggregate data by age, sex, ethnicity, race, caste, occupation, education, income, and employment status, where national law and context permit [20]. It is important to note that certain dimensions of inequality (such as race/ethnicity, caste, aboriginal status, migrant population, religion, and other minority status) may be of variable relevance, depending on the setting. While global monitoring of these dimensions may be impractical, national-level monitoring should be designed to incorporate relevant, setting-specific dimensions of inequality. With the advent of UHC on the global health agenda, experts and consultation groups have put forth recommendations for global inequality monitoring according to dimensions of inequality that can be measured comparably across countries, such as socioeconomic position, sex, geographical distribution, and other relevant factors [8],[16],[21].

**We recommend that global monitoring activities include complementary dimensions of inequality, such as economic status and urban/rural residence; sex should also be included.**

From a technical perspective, global monitoring of health inequality in UHC would ideally encompass four key dimensions of inequality: economic status, education, sex, and urban/rural residence. Consequently, a reasonably broad array of dimensions of inequality with high relevance—and data availability—is included at a global level.

Given that it may not be feasible for global monitoring to cover four different dimensions, economic status and urban/rural residence are appropriate choices for inclusion. Economic status is a valid and widely applied metric to show the distribution of health in a population, with established data collection systems and rigorous methodological study in diverse settings [22]. It is also opportune to consider urban/rural residence as a second dimension, as geographic-based dimensions of inequality offer clear, easily identifiable points for policy intervention. Economic status and urban/rural residence have wide applicability across all health indicators. For relevant indicators, sex should also be included in inequality monitoring as sex disaggregation is essential for efforts to promote gender equality. Education is an important factor as levels can be translated across countries.

## Formulating Subgroups

For a given dimension of inequality, formulating the criteria, number, and size of population subgroups is an important methodological consideration, with implications for monitoring. While some dimensions of inequality may seem to naturally present as straightforward subgroups (e.g., urban/rural residence or female/male sex), closer consideration may reveal significant ambiguity; other dimensions, such as economic status, require more arbitrary—albeit justified—classification of subgroups (see Box 1). The formulation of subgroups for global inequality monitoring must reflect classifications that have relevance across countries.

**Box 1. Metrics to Define Economic Status and Urban/Rural Residence**

Selecting metrics—that have applicability across countries—to define dimensions of inequality is subject to limitations. Challenges arise when attempting to construct a globally applicable common measure for economic status, as the constructs of economic status differ between high-income countries and low- and middle-income countries [22],[34]. Individual or household income is among the preferred metrics of economic positioning in high-income countries (where remuneration is more likely to be in monetary form, and received through formal employment), whereas household asset indices—reflecting ownership of durable goods and household characteristics—may be a more feasible measurement in low- and middle-income countries. Consumption data relate to the final use of goods and services, and are primarily obtained through collecting expenditure data [22]. This is the primary methodology used for international poverty monitoring.

In the case of urban/rural residence, the criteria used to differentiate urban and rural residents are not standardized across countries. In some situations, urban/rural residence classifications may be objective and well defined, although in others, substantial ambiguity may exist.

A major consideration when formulating subgroups pertains to subgroup heterogeneity. This issue may arise when subgroups are too expansive, masking important differences within. For example, monitoring efforts that are based on the poorest 40% may mask what is going on among the poorest of the poor; a situation where the poorest 20% have much lower coverage than the second poorest 20% would be concealed by only presenting data for the combined poorest 40%. The implications of masking the situation in the bottom quintile may stall progress on the goal of reducing the most stark health inequalities. Likewise, the rapid pace of urbanization, especially within low-income countries, has changed the nature of inequalities between and within urban and rural areas in many countries. With an increasing number of people residing in urban slums and informal settlements [23], health inequalities within urban environments themselves may constitute an important priority for monitoring within certain countries [24],[25]. For a given dimension of inequality, however, the extent of heterogeneity may vary between countries and between health indicators.

**We recommend that, for global monitoring, economic-related inequality be conceptualized using quintiles, and urban/rural residence conceptualized as a binary outcome.**

Wealth quintiles, which divide the population into five segments on the basis of economic status, can be easily communicated and understood by non-technical audiences, and have been widely applied in health reports. The use of quintiles to conceptualize economic-related inequality helps to alleviate the issue of masking.

While urban and rural categories might be further broken down at a national-level, binary classification offers a concise and acceptable depiction of area of residence, and can be applied across countries for global monitoring (recognizing that the national criteria used to define urban and rural subgroups may vary).

## Approaches to Monitoring Health Inequalities

Choices about how to frame and measure health inequalities have important implications for monitoring [26]; the conclusions about health inequality and interpretations of changes in inequality over time may differ on the basis of how a situation is conceptualized. There are three approaches that are commonly used to conceptualize and measure health inequalities: worst-off, gap, or whole spectrum (gradient) [27],[28].

The worst-off approach to monitoring focuses only on the situation in the most-disadvantaged subgroup, which is effectively unlinked from progress in other, more-advantaged subgroups. For example, a worst-off approach might report progress towards full coverage of a health service in the poorest 40% of a population where the initial coverage is lowest. This approach does not provide an indication of inequality between subgroups within a country, but rather a means by which to track the situation in the subgroup that is the most disadvantaged [17]. Without data from other subgroups, the worst-off approach may not provide important policy- and programme-relevant information required by Ministries of Health to ensure equity-oriented progressive realization of UHC.

Inequality is a relational concept; for this reason, true health inequality is conceptualized using gap and/or whole spectrum approaches that link the situation in a disadvantaged segment of the population with a more advantaged subgroup [27],[28]. The gap approach considers health differences between two subgroups. It can be applied to express inequality using dimensions of inequality that have only two categories, but also to demonstrate disparity between extreme groups (such as the richest 20% versus poorest 20%). In the progressive realization of UHC, the optimal situation is for the levelling up to complete coverage, with increased coverage in both the disadvantaged and advantaged subgroups, accompanied by a decreasing gap due to faster improvements in the disadvantaged group [29].

A whole spectrum approach applies to dimensions of inequality that contain multiple subgroups, and considers the situation across the entire population. Subgroups may have an inherent rank, as is the case with economic status or education, or be naturally unordered, as is the case with region or race. Although the distinction of whether subgroups are inherently ordered or unordered may imply different methods, inequality can be effectively expressed across dimensions of inequality that contain multiple subgroups.

**Figure 1:**
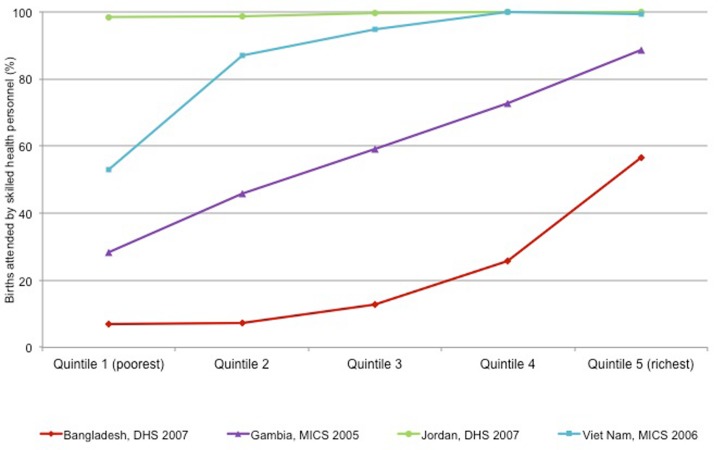
Patterns of inequality by wealth quintile, illustrated using births attended by skilled health personnel. Four characteristic patterns of inequality across household wealth quintiles for coverage of births attended by skilled health personnel in four countries are shown: Bangladesh (mass deprivation), Gambia (queuing), Jordan (complete coverage), and Viet Nam (marginal exclusion). Data were collected as part of Demographic and Health Surveys and Multiple Indicator Cluster Surveys, 2005–2007. Wealth quintiles were determined using household asset indices. Source: [18].

For dimensions of inequality that can be ranked, inequality across the social gradient can be effectively demonstrated by identifying patterns of inequality in disaggregated data. As illustrated using coverage of births attended by skilled health personnel in four countries, there are four characteristic patterns of inequality across social gradients, each suggesting a different policy response (Figure 1). In situations of mass deprivation, as demonstrated in Bangladesh, the disadvantaged group comprises a broad segment of the population, beyond the poorest 20% or even the poorest 40%. A pattern of mass deprivation may indicate a need for policies with a broad, population-wide focus. Health indicators that demonstrate marginal exclusion, as in Viet Nam, where disadvantage is experienced to a greater extent by a small proportion of the population, can be best addressed by primarily targeting the most disadvantaged subgroup. Queuing patterns (Gambia) require a combination of focusing on the population-at-large, with special targeting of the most disadvantaged. Finally, the pattern of complete coverage in Jordan requires on-going monitoring to ensure that this favourable situation is maintained. Examining the shapes of inequality across a gradient is an important aspect of national monitoring, revealing patterns in disaggregated data and generating evidence to support appropriate policy and targeting for the promotion of UHC [18].

**We recommend monitoring health inequality according to the gap approach, or alternatively, the whole spectrum (gradient) approach.**

We highlight the need to simultaneously consider the situation in at least two subgroups of the population, especially when tracking progress over time. This practice is particularly salient with regards to the progressive realization of UHC, as improvements should optimally be accelerated among disadvantaged groups.

Whereas a binary dimension of inequality, such as urban/rural residence or sex, can only be measured using a gap approach, inequality related to economic status could be monitored by either a gap approach (comparing two subgroups, such as the poorest and richest quintiles) or a whole spectrum approach (across the wealth spectrum). From a technical perspective, the whole spectrum approach offers a more nuanced and complete representation of inequality, allowing for more sophisticated presentation and measurement of inequality [18],[30]. A whole spectrum (gradient) approach considers information across the whole population, and thus allows a comprehensive appreciation of the shape of inequality across a population, generating evidence for targeting policies and initiatives aimed at reducing the social gradient in health [18],[27],[28]. The consistency of using a gap approach for economic-related, urban/rural residence, and sex-related inequality, however, is advantageous for conveying progress of global inequality monitoring.

## Measuring and Communicating Inequality

Measures of health inequality summarize disaggregated data from subgroups, expressing inequalities between subgroups in a concise manner. Measures of inequality can be rudimentarily classified as: (1) simple pairwise comparisons between two subgroups, or (2) complex comparisons based on data across multiple subgroups, used when adopting the gradient approach or when measuring inequalities between subgroups with no natural ordering [18],[30]. Some measures of inequality and the considerations for selecting them are outlined in Box 2.

**Box 2. Measures of Inequality**

The choice of an appropriate measure of inequality depends on the nature of the dimension of inequality (whether subgroups are ordered or not), and several other considerations like the desired point of reference (such as the overall average versus the best-performing subgroup) and whether the subgroups are weighted or unweighted (accounting for the population size of each subgroup, or treating subgroups as if they are equally sized, respectively). Whereas pairwise comparisons are generally unweighted, complex measures may be either unweighted or weighted. Although the interpretation of complex measures of inequality may be less intuitive than simple differences or ratios, complex measures have certain advantages over pairwise comparisons. Namely, they are useful to express inequality between differently sized subgroups, and can account for changing subgroup population sizes. When there are multiple subgroups, complex measures show inequality across the entire population.

Slope index of inequality and concentration index are two examples of complex measures that express inequality across a gradient of ordered subgroups, such as wealth quintiles or education levels [18],[35]. Slope index of inequality is an absolute measure, showing, for a given health indicator, the magnitude of difference in the whole spectrum, taking into account the mean value of health and population size in each subgroup. Concentration index is a relative measure of inequality, and expresses the extent to which the health indicator is concentrated among the disadvantaged or advantaged.

Other measures, such as the variance type measures or Theil index, can be applied to dimensions of inequality that have unordered subgroups, such as regions or racial/ethnic groups.

Population attributable risk is a measure of impact, based on the concept that inequality could be eliminated by all subgroups improving to the same level of health as the most advantaged or the best performing subgroup. Population attributable risk is a useful measure to show progress towards UHC [18],[36].

Certain health topics may prefer to use specific measures of inequality over others to suit a particular context [37].

Measures of inequality communicate either absolute or relative inequality. Absolute measures reflect the magnitude of inequality and retain the same or similar unit of measure as the health indicator, making the interpretation intuitive. Relative measures show proportional differences, and do not retain the unit of measure, making them useful for comparisons between health indicators with different units of measure. A major limitation of relative measures is the absence of information about the magnitude of difference. For example, a relative difference of two could represent coverage of 100% and 50% or 10% and 5%, which reflect very different absolute differences (50 percentage points compared to five percentage points). Together, measures of absolute and relative inequality inform a comprehensive understanding of health inequality, especially when reported alongside disaggregated data and national average.

**We recommend reporting both absolute and relative inequality; if reporting must be concise, we recommend emphasizing absolute inequality.**

Optimally, measures of absolute and relative inequality should be reported together to portray a more-complete understanding than either alone. For the gap approach, inequality is commonly measured as a difference (absolute measure of inequality) or a ratio (relative measure of inequality) between the two subgroups. These measures may be applied to binary stratifiers (showing inequality between urban and rural residents or females and males, for example), but also to dimensions of inequality with multiple subgroups (showing inequality between richest and poorest wealth quintiles, for example). For dimensions of inequality with multiple subgroups, absolute and relative inequality can also be measured using complex measures of inequality, which take into account the coverage across all subgroups.

When communication about health inequality must be concise, we recommend focusing on absolute inequality, as it provides an indication of magnitude of difference between subgroups and therefore may be simpler to conceptualize.

**We recommend that disaggregated data and summary measures of inequality are reported alongside the national average.**

Regardless of the approaches and measures chosen to monitor inequality, it is important to report UHC progress by both averages and inequality [18], which provides context for assessing the global situation more comprehensively. Inequality and national average reported together inform a more complete assessment of the situation than either in isolation.

## Target Setting

Ultimately, the goal of UHC is 100% coverage of essential health services with 100% financial protection, and by extension, elimination of associated inequality. Given the implementation of UHC through progressive realization, realistic equity-based global targets should take into account different baseline levels of inequality and national averages. Targets based on a proportional reduction were used for certain health-related MDG targets, which specified a proportional reduction of maternal and child mortality over 25 years [31] and thereby made the targets relevant to countries with variable initial levels of mortality.

**We recommend a target based on proportional reduction of absolute inequality.**

To emphasize changes in inequality over time and account for different baseline levels of inequality, we recommend a target specifying a proportional reduction in absolute inequality. Given that the baseline levels of inequality for different health indicators may vary substantially, this approach to target setting offers greater flexibility than a fixed absolute target across indicators. In addition, because the level of coverage that is feasible may be different for different types of health indicators, this approach alleviates the need to set multiple targets that are specific to categories of health indicators.

While it would be ideal to establish separate, complementary targets on the basis of absolute and relative inequality, we recognize that it may be more reasonable to advocate for inequality monitoring by focusing on one type of inequality. This recommendation is based on absolute inequality because it reflects the magnitude of difference. The six recommendations for global equity-oriented monitoring are summarized in Table 1.

**Table 1:** Summary of recommendations for global equity-oriented monitoring.

Recommendation	Basis	Technical Considerations and Limitations
**Global monitoring activities should include complementary dimensions of inequality, such as economic status and urban/rural residence; sex should be included.**	Inequality is multidimensional.	Ideally, global monitoring should include economic status, education, sex, and urban/rural residence.Dimensions may not be equally applicable across countries and health indicators.
**For global monitoring, economic-related inequality should be conceptualized using quintiles, and urban/rural residence, as a binary outcome.**	Heterogeneity exists within population subgroups.	Formulating subgroups by economic-related quintiles follows previous convention, and helps to alleviate masking issues.Formulating subgroups by urban/rural residence is intuitive and can be applied across countries.
**Global health inequality should be monitored using the gap approach or, alternatively, the whole spectrum approach.**	Inequality spans populations.	**F**or dimensions of inequality that are based on two subgroups (such as urban/rural residence), the gap approach is appropriate; for more than two subgroups (such as wealth quintiles), a whole spectrum approach can be used to express inequality across all subgroups.
**Report both absolute and relative inequality; if reporting must be concise, absolute inequality should be emphasized.**	Inequality is both an absolute and relative concept.	Absolute or relative measures used in isolation do not fully convey inequality, and thus should be reported together.Absolute inequality shows magnitude and may be more intuitive to understand than relative inequality.
**Monitoring of inequalities should be reported alongside an indication of national average.**	Monitoring health inequalities along with national average provide a fuller context.	When comparing a group of countries, presenting the median value may be appropriate for both inequality and national average.
**Targets should be based on proportional reduction of absolute inequality.**	The baseline level of inequality for different health services may vary substantially.	Ideally, targets should convey both absolute and relative inequality.When setting targets for changes in inequality over time, targets should specify a proportional reduction in absolute inequality.

An empirical-based example uses household survey data from 1993 to 2011 to demonstrate the application and reporting of these six recommendations in low- and middle-income countries (Box 3). Median coverage for six health services showed reductions in absolute inequality ranging from 17.9% to 49.3% for economic-related inequality (at least four antenatal care visits and DTP3 immunization, respectively) and 25.5% to 54.7% for urban/rural inequality (births attended by skilled health personnel and DTP3 immunization, respectively), over a 10 year period (Table 2).

**Table 2:** Application of recommendations for target-setting for global monitoring of economic-related and urban/rural residence inequality in health.

Health Indicator	Economic-Related Absolute Inequality at Baseline (Percentage Points)	Reduction of Economic-Related Absolute Inequality over 10 Years	Urban/Rural Absolute Inequality at Baseline (Percentage Points)	Reduction of Urban/Rural Absolute Inequality over 10 Years	Median Overall Coverage at Baseline	Median Increase in Coverage over 10 Years
**Family planning needs satisfied**	23.0	44.0%	14.7	49.5%	60.9%	7.6%
**Antenatal care (at least one visit)**	26.3	40.6%	11.4	49.4%	80.5%	8.1%
**Antenatal care (at least four visits)**	32.2	17.9%	22.3	34.7%	51.5%	22.2%
**Births attended by skilled health personnel**	53.0	19.7%	30.5	25.5%	48.4%	13.9%
**DTP3 immunization**	23.2	49.3%	10.8	54.7%	72.1%	17.3%
**Measles immunization**	20.8	45.2%	9.9	47.8%	73.9%	11.7%

Relevant data for the application of targets for global monitoring of health service coverage, applied to six reproductive, maternal, and child health service indicators in 26–31 countries are shown. Absolute inequality at baseline was calculated as the median difference in coverage between the richest and poorest quintiles (quintiles determined using household asset indices), or between urban and rural areas. The reduction of absolute inequality over 10 years was calculated as the median relative change in absolute difference in coverage between the richest and poorest quintiles, or between urban and rural areas, over a 10 year interval. The median overall coverage and median increase in coverage are displayed alongside. Data were collected as part of Demographic and Health Surveys and Multiple Indicator Cluster Surveys. Because survey years were not consistent across countries, country-level data spanning 9–11 year intervals were collected at two time points between 1993–2011.

**Box 3. Applying a Target Based on Proportional Reduction of Absolute Inequality, and Reporting Progress over Time: An Empirical Example**

Based on previous progress measured across various reproductive, maternal, and child health service indicators, we specified sample targets for global monitoring of health inequality:

- Achieve a 50% reduction in the absolute difference in health service coverage between the poorest 20% and the richest 20%.
- Achieve a 50% reduction in the absolute difference in health service coverage between urban and rural areas.

**Figure 2:**
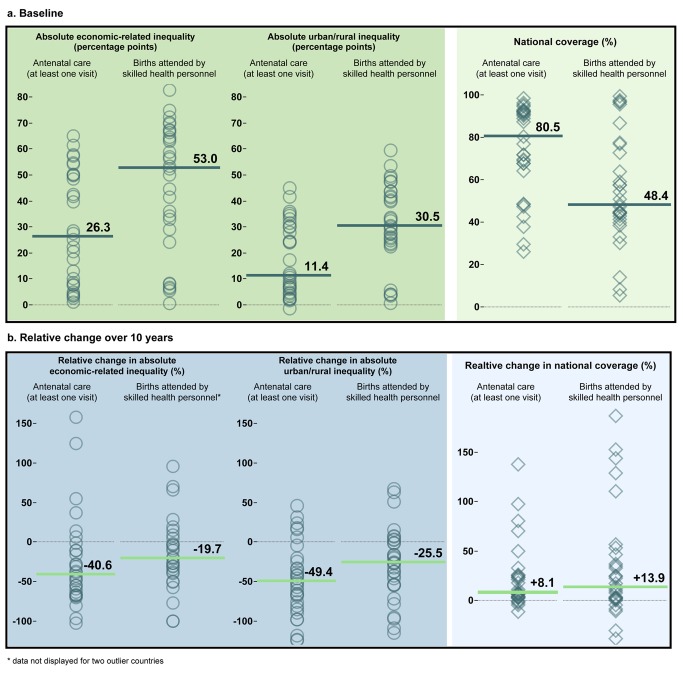
Visualization of sample targets for global inequality monitoring of health service coverage. A visualization of two sample targets for global monitoring of health service coverage (Box 3), applied to antenatal care (at least one visit) and births attended by skilled health personnel, in 30–31 countries. (a) Absolute inequality at baseline between the richest and poorest quintiles (quintiles determined using household asset indices), and urban and rural areas, along with overall national coverage at baseline; (b) the relative change in absolute inequality over 10 years, along with the relative change in national coverage. Shapes represent countries; within each pane, each country is represented by one shape. Horizontal lines indicate median values of all countries within the pane. Data were collected as part of Demographic and Health Surveys and Multiple Indicator Cluster Surveys. Because survey years were not consistent across countries, country-level data spanning 9–11 year intervals were drawn from surveys at two time points between 1993–2011.

In accordance with the proposed recommendations for global monitoring of inequality, these two sample targets were applied to empirical data from household surveys conducted over the period from 1993 to 2011, looking at coverage of antenatal care (at least one visit) and births attended by skilled health personnel in 30–31 low- and middle-income countries. Figure 2 is a visual representation of how such a target could be presented, using median values derived from the multiple study countries to report inequality and national average of health service coverage. (We emphasize that this exercise is intended to exemplify a sample target and how progress may be measured and communicated; the process of setting targets for global monitoring in UHC will necessitate more extensive exploration of empirical data, as well as consultation with experts and country representatives.)

Median absolute inequality at baseline was lower for antenatal care than births attended by skilled health personnel for both economic related inequality (26.3 and 53.0 percentage points, respectively), and urban/rural inequality (11.4 and 30.5 percentage points, respectively); the median national coverage at baseline, reported alongside inequality, was 80.5% for at least one antenatal care visit and 48.4% for births attended by skilled health personnel (Figure 2a).

Figure 2b shows the median reduction in inequality over 10 years, which ranged from a minimum of 19.7% reduction for economic-related inequality in coverage of births attended by skilled health personnel, to a maximum of 49.4% reduction in urban/rural inequality in coverage of at least one antenatal care visit. The relative increases in median overall coverage were 8.1% for at least one antenatal care visit and 13.9% for births attended by skilled health personnel, respectively.

Considering these results in the context of the sample target that specifies a 50% reduction, antenatal care coverage is closer to meeting the targets than coverage of births attended by skilled health personnel: a 40%–50% reduction in both inequality dimensions over 10 years has been achieved in antenatal care coverage. A great deal of progress would have to be made for coverage of births attended by skilled health personnel to achieve this target, as inequalities were reduced by about 20%–25% over the 10 year period.

## Steps Forward

Looking ahead to post-2015, building capacity for health inequality monitoring at both global and national levels is timely, relevant, and important. A country's capacity to conduct health inequality monitoring is largely determined by the performance of the health information system that collects data, analyzes data, reports inequality, and disseminates results. Although this paper has primarily explored considerations for global health inequality monitoring, strengthening a country's capacity to conduct health inequality monitoring will concurrently facilitate improvements and expansion in national inequality monitoring efforts.

Initiatives are warranted to improve the collection, quality, and use of data for health inequality monitoring (ensuring that necessary data protection mechanisms are established [32]) and develop the technical proficiency to conduct regular health inequality monitoring. The strength of a country's health information system has implications for inequality monitoring as it provides data inputs for comparisons of population subgroups. Good quality and comparable data may not be available across a number of countries and/or health indicators, especially with respect to baseline data. For example, household health examination surveys containing noncommunicable disease indicators are lacking in many low- and middle-income countries; while the WHO Stepwise approach has made headway towards this aim [33], substantial investments would be required to expand and strengthen international surveys with comparable data and methodology. (For more information about data sources please see Box 4.)

**Box 4. Data Sources**

The main data sources for monitoring inequality in UHC are household surveys as well as facility records. Household surveys are the best available data source for global- and country-level inequality monitoring, and generally provide rich data on the two main components of UHC (access to health services and financial risk protection), and many dimensions of inequality [18],[37]. Household surveys are a population-based source of information, containing data on a representative sample of the population. Facility records include data gathered in the course of administrative and operational activities, and are limited to individuals that interact with the given institution. Such institution-based records may provide data that are unstandardized across facilities and highly fragmented in countries that have not made efforts towards harmonization.

Sourcing reliable data for health inequality monitoring may pose a challenge, especially in many low- and middle-income countries. Countries may strengthen data sources—and thus increase capacity for health inequality monitoring—through efforts to: expand and conduct regular, recurring household surveys, optimally every few years in all countries [37]; and harmonize data collected through facilities through means such as standardizing electronic records across institutions. Additionally, the utility of data sources could be improved by incorporating individual or small-area identifiers (such as social security numbers or postal codes) to enable linking between sources and improve the ability to disaggregate data. For example, census data about a dimension of inequality like economic status may be linked to facility data specific to health service coverage.

Once there are data available that are suitable for inequality analyses, expertise is required to perform these analyses and communicate the results. To have impact and appeal to a range of stakeholders (including policy makers and the general public), global inequality monitoring should be as straightforward and coherent as possible. Visualization technology can facilitate the presentation and interpretation of dense inequality datasets, as results may be displayed interactively and/or simultaneously across many countries [4]. Sometimes the clear and effective communication of multiple dimensions of inequality may necessitate a reduction in the amount of data that are presented. Reporting inequalities may require careful negotiation to strike a balance between presenting a simple, comprehensible message, yet maintaining a sufficient degree of richness and rigor to ensure that the results are communicated accurately. When this accuracy is achieved, health inequality monitoring can meaningfully inform efforts to improve both overall coverage and equity-oriented progress towards UHC. For some countries, significant investments may be required to build capacity for health inequality monitoring.

## Conclusions

Discussions on the post-2015 global health agenda, including those in relation to UHC, present a timely and appropriate opportunity to mainstream the practice of health inequality monitoring on a global scale. The promotion of equity-oriented global monitoring of UHC entails establishing a unified approach to monitoring that enables global comparisons between countries (i.e., cross-country comparisons of within-country inequalities). Moving forward, ensuring conceptual and technical precision while garnering widespread appeal and adoptability will be key challenges in establishing a protocol for global health inequality monitoring.

## Abbreviations

UHC: universal health coverage
